# Measured vs self-reported overweight/obesity in the Italian adult population: CUORE Project 2018-19

**DOI:** 10.1093/eurpub/ckac131.556

**Published:** 2022-10-25

**Authors:** C Donfrancesco, E Bologna, L Iannucci, E Profumo, C Lo Noce, B Buttari, A Di Lonardo, S Vannucchi, G Onder, L Palmieri

**Affiliations:** 1 Istituto Superiore di Sanità, Rome, Italy; 2 Italian National Institute of Statistics, Rome, Italy

## Abstract

**Background:**

In monitoring population health and the effectiveness of public health strategies, the body mass index (BMI) is often assessed within national surveys from self-reported height and weight rather than measured values. Using data collected within a recent health examination survey (HES), the discrepancies between self-reported and measured values were assessed, and correction models were estimated and implemented on national interview survey data.

**Methods:**

Within the CUORE Project, the Italian National Institute of Health conducted the HES 2018-2019 measuring height and weight as well as collecting data on self-reported values in random samples of general population aged 35-74 years residing in ten (of 20) Italian regions distributed in the North, Centre and South: 1033 men and 1061 women.

**Results:**

Self-reported and measured data comparison showed greater differences in mean values of height than weight and in women than in men (height +2 cm in men and +3.2 in women; weight -0.7 kg and -1.4 kg, respectively) and a corresponding underestimation of BMI (-0.7 kg/m2 and -1.4 kg/m2, respectively). Differences were stable across age groups and educational levels, except for height discrepancy, which was greatest in women aged 65-74 years. Self-reported vs measured prevalence were: normal weight 39.7%-33.3% in men and 54.8%-44.7% in women, overweight 45.8%-46.1% and 26.0%-29.2%, obesity 13.8%-20.1% and 15.7%-23.9%. Linear regression models adjusted by sex and age classes were assessed for height and weight (R2 > =0.92) and implemented to estimate adjusted BMI and normal weight/overweight/obesity prevalence on the national multi-purpose interview survey data collected by the Italian National Institute of Statistics.

**Conclusions:**

To provide more accurate prevalence of normal weight, overweight and obesity, self-reported values could be adjusted using correction models developed on the basis of the relationship between self-reported and measured height and weight values.

**Key messages:**

• Discrepancies between self-reported and measured values of height and weoght were found.

• Self-reported values could be adjusted using correction models developed on the basis of the relationship between self-reported and measured height and weight values.

